# Recurrent Acute-on-Chronic Pancreatitis in a Chronic Alcoholic With Pancreatic Divisum: A Complex Case

**DOI:** 10.7759/cureus.53022

**Published:** 2024-01-26

**Authors:** Sarang S Raut, Sourya Acharya, Sunil Kumar, Vinit Deolikar, Manjeet Kothari

**Affiliations:** 1 General Medicine, Jawaharlal Nehru Medical College, Wardha, IND

**Keywords:** pancreatic lithotripsy, endoscopic retrograde cholangiopancreatography, recurrent acute pancreatitis, chronic calcific pancreatitis, pancreatic divisum

## Abstract

Pancreatic divisum, a rare developmental anomaly of the pancreas, is infrequently observed in young individuals and is a recognized cause of recurrent acute pancreatitis, ultimately progressing to chronic pancreatitis. Chronic alcoholism is a prevalent and significant etiological factor contributing to both recurrent acute pancreatitis and chronic pancreatitis. This case report details the presentation of a 28-year-old patient with a history of chronic alcoholism, exhibiting recurrent acute pancreatitis evolving into chronic pancreatitis. Diagnostic evaluation revealed the presence of pancreatic divisum with associated pancreatic intraductal calculi, a common trigger for pancreatitis. The patient underwent successful management through endoscopic retrograde cholangiopancreatography, pancreatic lithotripsy, and pigtail pancreatic stent placement. This case highlights the complex interplay between chronic alcoholism leading to recurrent acute pancreatitis and pancreatic divisum, a rare congenital anomaly, which is found later in this case, complicating the disease.

## Introduction

With a prevalence of 4% to 14% of the population, pancreatic divisum is one of the rare developmental anomalies in which the ventral and dorsal buds of the organ do not fuse. The dorsal and ventral pancreas fuse together by rotating the pancreatic ventral bud around the foregut during normal embryological development. The ventral and dorsal pancreatic ducts unite to form the main pancreatic duct, also referred to as the Wirsung duct. This duct then joins the common bile duct and empties into the major duodenal papillae. This primary pancreatic duct is where most of the typical pancreata empty. The minor papilla allows the dorsal duct to empty into the duodenum, where it continues as the Santorini duct. Conversely, pancreatic divisum happens when the dorsal and ventral buds are unable to unite [1]. Pancreatic divisum is classified into three main categories. The complete failure of the dorsal and ventral buds to merge is known as type I or classic pancreatic divisum. The major papilla drains a portion of the common bile duct, while the minor papilla drains the entire pancreas in a type II pancreatic divisum, which is distinguished by the absence of the ventral duct. Lastly, type III exhibits a tiny residual ventral duct and dorsal duct connection [1]. Numerous predisposing factors contribute to the development of acute, recurrent, and chronic pancreatitis, including chronic alcoholism, dyslipidemia, and exposure to toxins, and among them is pancreatic divisum, a rare congenital anomaly that increases the susceptibility to both acute and chronic pancreatitis [2]. Some people with complete or incomplete pancreas divisum may need the existence of another cause, such as alcohol misuse, for chronic pancreatitis to develop, as is in our case [3].

Acute pancreatitis is an immediate response to damage inflicted upon the pancreas. On the other hand, chronic pancreatitis can cause enduring harm to the pancreas' structure and its endocrine and exocrine functionalities. The onset of acute pancreatitis can be attributed to mechanisms such as injury to the pancreatic duct and acinar regions. In this condition, the pancreas fails to properly secrete digestive enzymes, resulting in self-digestion and inflammation. Chronic pancreatitis, on the other hand, may develop through recurrent acute episodes, leading to the infiltration of inflammatory elements and the formation of fibrous tissue within the pancreas. This prolonged process eventually results in pancreatic insufficiency. Acute pancreatitis presents with sharp abdominal pain in the epigastric region radiating to the back, nausea, and vomiting. Chronic pancreatitis presents with abdominal pain, nausea, and vomiting. However, it can also present without abdominal pain but with steatorrhea, and weight loss [4].

Pancreatic divisum as a common etiology in young populations for acute recurrent pancreatitis and chronic pancreatitis should be considered and its complications should be managed unless it can lead to serious complications like acute necrosis of the pancreas and pseudocyst of the pancreas [5]. Also, pancreatitis can lead to a pancreaticocutaneous fistula [6]. Also, long-term alcohol consumption is a common cause of chronic pancreatitis progression and can lead to complications like pseudocyst of the pancreas, pancreatic calculi, and biliary dilatation [7]. A pseudocyst of the pancreas can get complicated as a mediastinal extension, which is a rare and deadly complication and its timely diagnosis can prevent mortality [8]. Pylephlebitis is thrombophlebitis of the portal vein or its hepatic branches with positive blood culture. Pylephlebitis and hepatic abscesses are the startling complications of intraabdominal sepsis and if present in patients with acute pancreatitis can also complicate the ongoing acute pancreatitis [9].

During endoscopic retrograde cholangiopancreatography, which is a combined endoscopic procedure, an endoscope is advanced into the second portion of the duodenum. This makes it easier for other instruments to enter the pancreatic and biliary ducts through the major duodenal papilla. When necessary, therapeutic intervention and radiologic visualization can be made possible by injecting contrast material into these ducts. Because of the cannulation of the pancreatic and biliary ducts, endoscopic retrograde cholangiopancreatography (ERCP) has developed into a diagnostic and therapeutic tool [10]. In our case, chronic calcific pancreatitis was suspected based on an ultrasound abdomen-pelvis. ERCP was done which revealed pancreatic divisum along with chronic calcific pancreatitis with multiple radio-opaque calculi in the head of the pancreas and was managed successfully with pancreatic lithotripsy and pigtail pancreatic stent placement in minor papilla via ERCP.

## Case presentation

Over the past eight days, a 28-year-old man with a 12-year history of chronic alcoholism with a daily intake of 90 grams to 120 grams of alcohol approximately presented with intense abdominal pain in the epigastric area radiating to the back for the last eight days. The patient had no known comorbidities. The patient had a history of hospital admissions several times in the past two years due to acute pancreatitis. Upon general examination, the patient's overall condition was moderate. He was afebrile, with a pulse rate of 90 beats per minute. Notably, there were no signs of pallor, icterus, clubbing, cyanosis, lymphadenopathy, or pedal edema. In the systemic examination, abdominal examination revealed tenderness in the epigastrium with no organomegaly on palpation, both heart sounds were heard without murmurs on the cardiovascular examination. A respiratory system examination revealed bilateral air entry equal without adventitious sounds. The central nervous system examination confirmed the patient's consciousness and orientation to time, place, and person. Laboratory blood investigations are shown in Table 1. The patient had elevated serum lipase levels suggestive of pancreatic injury.

**Table 1:** Laboratory blood investigations of the patient

Lab parameters	Observed values	Normal range
Hemoglobin	12.9 g%	13-17 g%
Total leucocyte counts	17400 cell/mm^3^	4000-11000 cell/mm^3^
Platelets	2.35 cell/mm^3^	150000-400000 cell/mm^3^
Mean corpuscular volume	82 fL	83 - 101 fL
Serum creatinine	0.8 mg/dl	0.66 - 1.25 mg/dl
Serum urea	17 mg/dl	19 - 43 mg/dl
Serum sodium	139 mmol/L	137-145 mmol/L
Serum magnesium	1.7 mg/dl	1.6 - 2.3 mg/dl
Serum calcium	8.2 mg/dl	8.4 - 10.2 mg/dl
Serum potassium	3.7 mmol/L	3.5-5.1 mmol/L
Serum alkaline phosphatase	116 U/L	38-126 U/L
Serum aspartate transaminase	21 U/L	17-59 U/L
Serum alanine transaminase	24 U/L	<50 U/L
Serum albumin	4.4 g/dL	3.5-5 g/dL
Serum total bilirubin	0.6 mg/dL	0.2-1.3 mg/dL
Serum lipase	1000 U/L	23 - 300 U/L
Serum amylase	700 U/L	30 - 110 U/L
International normalized ratio	1	< 1.1

An ultrasound of the abdomen and pelvis was performed as part of the diagnostic process, and the results pointed to a possible diagnosis of chronic calcific pancreatitis. Notably, intraductal calculi were found in the pancreatic head and neck area. The diagnosis was confirmed by a follow-up contrast-enhanced CT scan of the abdomen, which showed multiple radio-opaque calculi in the head and neck area and a dilated pancreatic duct with chronic calcific pancreatitis.

For further management, an endoscopic retrograde cholangiopancreatography (ERCP) was performed using an EVIS JF 180 scope (Olympus Corporation, Shinjuku City, Tokyo, Japan. The ERCP findings indicated pancreatic divisum with chronic calcific pancreatitis with intraductal calculi in the head region of the pancreas. The procedure involved cannulation of the pancreatic duct via the minor papilla, dilation of the pancreatic duct, sphincterotomy of the minor papilla, pancreatic lithotripsy, and flushing of stone fragments with normal saline. Additionally, a 7Fr 7 cm single pigtail pancreatic stent was placed via the minor papilla.

**Figure 1:**
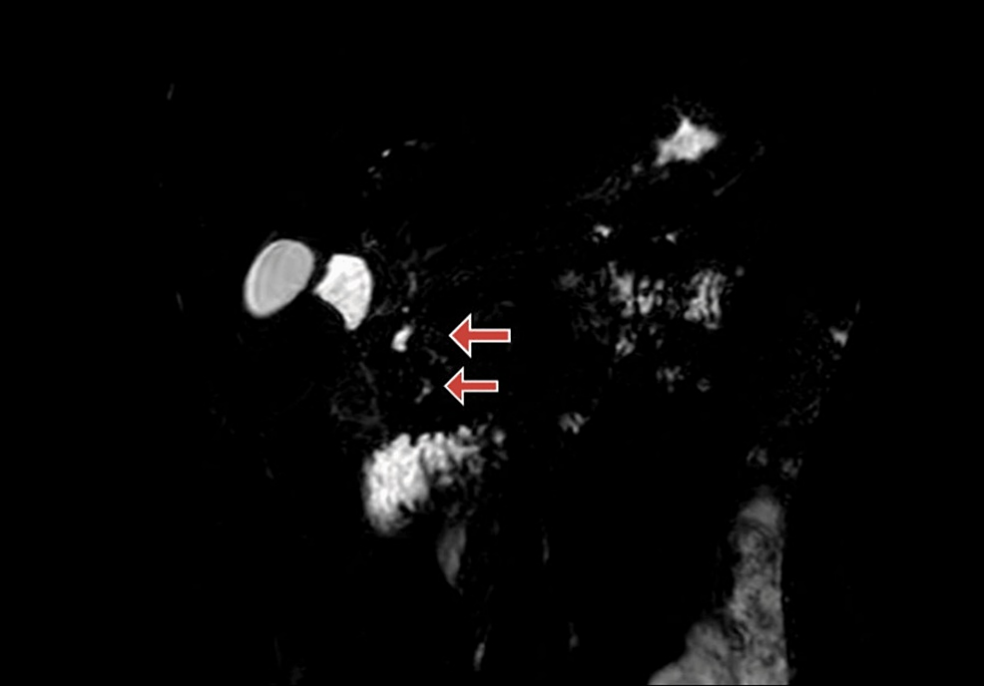
Magnetic resonance cholangiopancreatography (MRCP) high-resolution 3D reconstruction image showing pancreatic divisum (red arrow).

**Figure 2:**
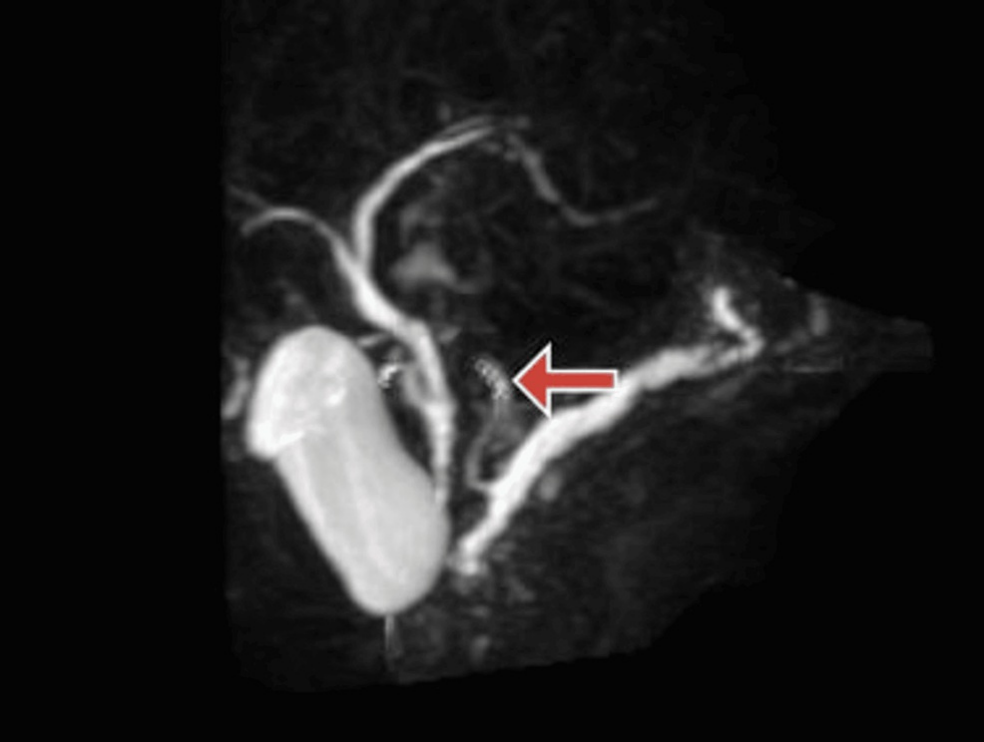
Magnetic resonance cholangiopancreatography (MRCP) 3D reconstruction image showing pancreatic divisum with a stent placed in a minor papilla (red arrow).

**Figure 3:**
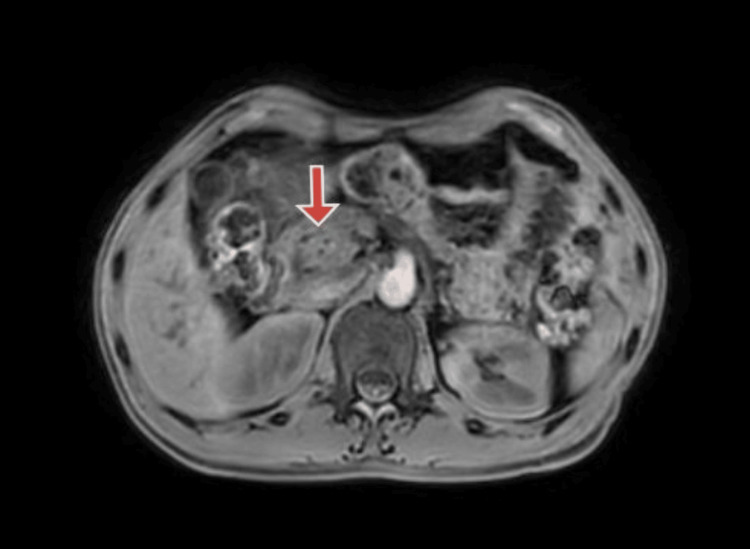
Magnetic resonance cholangiopancreatography (MRCP) 3D VANE XD image showing calcification in pancreas suggestive of chronic calcific pancreatitis (red arrow).

**Figure 4:**
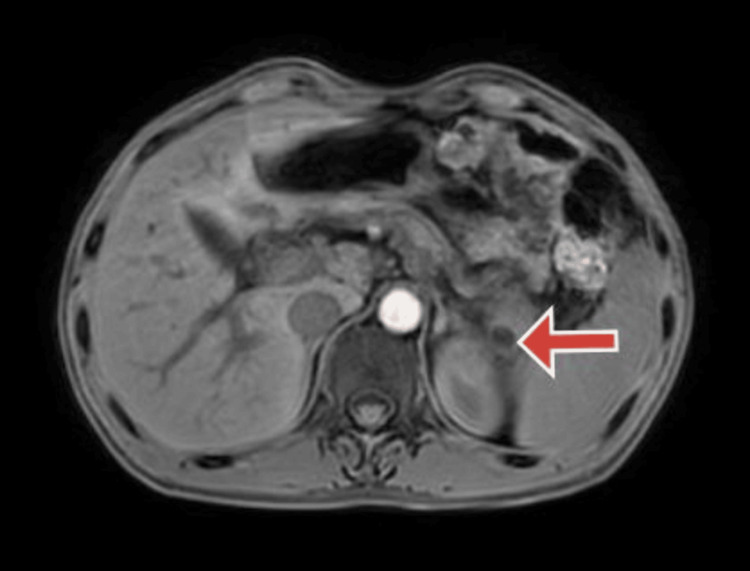
Magnetic resonance cholangiopancreatography (MRCP) 3D VANE XD image showing a small pseudocyst in the tail region of the pancreas (red arrow).

A month later follow-up ultrasound of the abdomen and pelvis revealed multiple calcifications within the pancreas, indicative of ongoing chronic calcific pancreatitis with a stent in the minor papilla. Subsequent magnetic resonance cholangiopancreatography (MRCP) aimed to examine the pancreaticobiliary system as a follow-up. The MRCP findings suggested pancreatic divisum with changes consistent with chronic pancreatitis and the development of a small pseudocyst in the tail region. Figure 1 shows an MRCP image of pancreatic divisum. Figure 2 shows an MRCP image showing the pancreatic divisum with a stent placed in a minor papilla. Figure 3 shows an MRCP image showing chronic calcified pancreatitis. Figure 4 shows an MRCP image showing a small pseudocyst in the tail region of the pancreas as a complication of chronic calcified pancreatitis.

In summary, this systematic case presentation outlines the clinical history, examination findings, diagnostic imaging results, interventions performed during ERCP, and subsequent follow-up observations, providing a comprehensive understanding of the patient's complex condition.

## Discussion

This case report underscores the intricate interplay between chronic alcoholism, pancreatic divisum, and recurrent acute pancreatitis, leading to the development of chronic calcific pancreatitis and associated complications. The patient's history of chronic alcohol misuse served as a significant predisposing factor, contributing to the recurrent nature of acute pancreatitis and ultimately progressing to chronic calcific pancreatitis.

The identification of pancreatic divisum in this case adds a layer of complexity to the understanding of the pathophysiology. Pancreatic divisum, a rare congenital anomaly, is recognized as a potential risk factor for both acute and chronic pancreatitis. Its classification into three types, with variations in ductal drainage patterns, further emphasizes the diverse anatomical presentations and clinical implications [11]. The recurrent hospitalizations for acute pancreatitis over the past two years indicate the challenges in managing such cases. Pancreatic divisum alone may not always lead to pancreatitis, but in combination with other factors, such as chronic alcohol consumption, it becomes a crucial contributor [12]. The role of alcohol in exacerbating pancreatic damage is well-established, and its association with chronic pancreatitis progression is evident in this case [13].

The diagnostic strategy, which included ERCP, contrast-enhanced CT, and ultrasound, was essential in determining the presence of pancreatic divisum, chronic calcific pancreatitis, and related complications. The pancreatic and biliary ducts could be seen, cannulated, and treated with the help of ERCP, which is both therapeutic and diagnostic [14]. The initial ERCP intervention, involving pancreatic duct cannulation, lithotripsy, sphincterotomy, and pancreatic stent placement, aimed at addressing the calculi and improving ductal drainage by placing a stent in a minor papilla. However, the subsequent presentation with the discovery of a pseudocyst on MRCP, which was done as a follow-up imaging, underscores the challenges in the long-term management of such cases. Pseudocyst formation is a recognized complication of chronic pancreatitis, and its occurrence highlights the need for ongoing surveillance and intervention [15].

## Conclusions

This case report highlights the significance of treating patients with recurrent acute-on-chronic pancreatitis due to chronic alcoholism with pancreatic divisum complicating the disease as it is a rare but important predisposing factor in the etiology of acute or chronic pancreatitis holistically, especially when alcohol abuse is present. To manage recurrent complications of acute-on-chronic pancreatitis and improve patient outcomes, routine imaging and long-term follow-ups are crucial with a multidisciplinary approach in cases of pancreatic divisum.
